# Overlap of traditional bullying and cyberbullying and correlates of bullying among Taiwanese adolescents: a cross-sectional study

**DOI:** 10.1186/s12889-019-8116-z

**Published:** 2019-12-30

**Authors:** Chia-Wen Wang, Patou Masika Musumari, Teeranee Techasrivichien, S. Pilar Suguimoto, Yukiko Tateyama, Chang-Chuan Chan, Masako Ono-Kihara, Masahiro Kihara, Takeo Nakayama

**Affiliations:** 1 Department of Health Informatics, Kyoto University School of Public Health, Yoshida Konoe-Cho, Sakyo-Ku, Kyoto, 606-8501 Japan; 2 Interdisciplinary Unit for Global Health, Centre for the Promotion of Interdisciplinary Education and Research, Kyoto University, Yoshida Hon-Machi, Sakyo-Ku, Kyoto, 606-8317 Japan; 3 Medical Education Centre, Kyoto University Graduate School of Medicine, Yoshida Konoe-Cho, Sakyo-Ku, Kyoto, 606-8501 Japan; 4 Kyoto University Health Service, Yoshida-Hon-Machi, Sakyo-Ku, Kyoto, 606-8501 Japan; 5 Institute of Occupational Medicine and Industrial Hygiene, College of Public Health, National Taiwan University, No.17, Xu-Zhou Rd., Taipei, 10055 Taiwan

**Keywords:** Traditional bullying, Cyberbullying, Combined bullying, Prevalence, Overlap, Risk factors

## Abstract

**Background:**

Due to the rapid development of information and communication technologies, cyberbullying has emerged as a threat to adolescents. This study aimed to investigate the prevalence and correlates among profiles of traditional bullying, cyberbullying, and combined bullying among Taiwanese high school students.

**Methods:**

This cross-sectional study employed two-stage cluster sampling in Taipei City, Taiwan. In total, 2028 high school students completed an anonymous questionnaire between March and May 2018. Nominal logistic regression analysis was performed, adjusting for clustering, to examine the correlates of each type-role category of bullying.

**Results:**

The prevalence rates of cyberbullying, traditional bullying, and combined bullying were 9.9, 13.3, and 9.4%, respectively, indicating that one-third of students were involved in one of these types of bullying; 48.7% of those involved in cyberbullying also experienced traditional bullying, and 41.5% of those involved in traditional bullying also experienced cyberbullying. In any type of bullying, not only being a victim but also being a bully/bully-victim was significantly associated with at least one mental health problem (serious psychological distress, self-harm, or suicidal ideation), except in the case of cyberbullying bullies/bully-victims. Internet abuse and alcohol use were more concentrated among bullies/bully-victims than victims for all types of bullying, and a similar trend was observed among types of schools and school climates, suggesting that specific behavioural circumstances or school backgrounds are associated with bullying perpetration.

**Conclusions:**

Bullying is a prevalent and complex phenomenon among adolescents in Taiwan, where traditional bullying and cyberbullying frequently overlap and are likely to occur against specific backgrounds. These facts should be taken into account in future bullying prevention and support programmes in Taiwan.

## Background

Bullying is a significant public health concern affecting the well-being of adolescents. Currently, as many as 246 million adolescents are estimated to suffer from school violence or bullying worldwide [1]. Bullying has a wide range of adverse health impacts for adolescents, such as depression [2], suicidal ideation, and suicidal behaviours [3].

With the rapid development and dissemination of information and communication technologies (ICTs), many adolescents use the internet and social networking services (SNSs) [4], which increases the opportunities for cyberbullying [5, 6]. Cyberbullying is defined as “bullying and harassment of others by means of new electronic technologies, primarily mobile phones and the internet” [7]. Compared with traditional bullying, cyberbullying has a unique nature with respect to publicity, anonymity, and the lack of supervision [8], which can lead to substantial psychological and psychiatric problems among victims [9, 10]. Furthermore, cyberbullying sometimes overlaps with traditional bullying and potentially leaves profound health impacts on victims. Previous studies have documented that adolescents exposed to two types of bullying are likely to suffer from higher levels of psychological distress than those involved in a single type of bullying [9, 11].

To date, most cyberbullying studies have been performed in Western societies and investigated cyberbullying’s prevalence [12, 13], its overlap with traditional bullying [9, 14–19], and the risk factors associated with it [13, 20, 21]. However, the results are inconsistent regarding the extent of overlap between cyberbullying and traditional bullying: some studies [9, 14, 15, 19] indicated wide-ranging overlap, while others [17, 18, 22] reported a more limited overlap. Although cyberbullying is a complex social phenomenon, few studies have attempted to understand its correlates from an ecological point of view [21, 23].

Bullying is an issue of interpersonal relationships. Since Western and Asian societies differ in the nature of interpersonal relationships, as represented by individualism and collectivism, respectively, it is possible that the nature of cyberbullying may also differ between these two societies. However, cyberbullying studies have only recently started to be conducted in Asian countries, such as China [24], Hong Kong [25], and South Korea [26], and most are concentrated within the last five years. In Taiwan, only one up-to-date study on the relationship between cyberbullying and traditional bullying was conducted, in 2010 [27].

Given this background and the explosive expansion of SNS use among Taiwanese adolescents in the last decade, this study aims to update the information on cyberbullying among high school students in Taiwan. It pays special attention to classifying bullying into traditional bullying, cyberbullying, or a combination of the two and comparing the correlate profiles from ecological points of view. These issues have never been explored in Asia, including Taiwan.

## Methods

### Study design, setting and participants

A cross-sectional study design was adopted. The target population consisted of high school students currently enrolled in grades 10 and 11 in Taipei City, Taiwan. Two-stage cluster sampling was performed. In the first stage, 30 high schools were randomly selected from a list of 67 high schools, and 22 schools (73.3%) decided to participate. Non-participation reasons were no responses and time constraints.

In the second stage, 2 classes were randomly selected from grades 10 and 11 of each school, and 3270 students were eligible to participate in this study. The number of attending participants between March and May in 2018 determined the sample size.

### Data collection

An anonymous paper-based questionnaire was used for data collection. Because of the sensitivity of the research topic, the participants were asked to complete the anonymous paper-based questionnaire at home to protect their privacy and avoid peer pressure. The structured questionnaire included the following variables: (1) bullying: traditional bullying and cyberbullying; (2) individual factors: demographic factors, academic level, internet usage time, internet addiction, substance use, and psychological and psychiatric factors; (3) family factors: living situation, parental internet supervision, and number of days eating dinner with family per week; and (4) school/social factors: type of school, school climate, and perceived social support. In this study, traditional and cyberbullying were dependent variables, and the remaining variables were independent variables.

### Measures

#### Survey instrument

The questionnaire in this study was developed based on the results of our prior qualitative study in 2016 [28] as well as a careful review of the international literature that included Taiwan [12, 27, 29–36] (the detailed questionnaire please see Additional file 1). We conducted two pilot studies (unpublished works) to improve the reliability and face validity of the questionnaire. The first phase pilot study was conducted in two high schools with 58 student participants recruited through convenience sampling in June 2017. Since some questions showed poor reliability and were found to be difficult to understand, we modified the questionnaire and tested it in the second phase of the pilot study with 89 students from two high schools recruited through convenience sampling from October to November 2017. This study showed that the test-retest reliability values (1-week interval) of the questionnaire measured by means of intraclass correlation coefficients (ICCs) were 0.48 and 0.75 for the victimisation and perpetration of traditional bullying, respectively, and 0.54 and 0.60 for the victimisation and perpetration of cyberbullying. In addition, other study variables showed Kappa coefficients between 0.51 and 1.00 for categorical variables and ICCs between 0.75 and 0.93 for continuous variables. These results demonstrated moderate to excellent reliability of the questionnaire [37, 38].

#### Traditional bullying

The revised Olweus Bully/Victim Questionnaire [29] was adopted to measure both victimisation and perpetration of traditional bullying. The participants were first asked how frequently they had experienced traditional victimisation in the previous 2 months. The following questions were included: “How often have other student(s) (1) called you mean names, made fun of you or teased you in a hurtful way; (2) excluded you from their group of friends or completely ignored you; (3) hit, kicked, pushed or shoved you; or (4) told lies or spread false rumours about you and tried to make others dislike you?” Then, the participants were asked how often they had perpetrated the above behaviours towards others. Each item was evaluated on a 5-point scale as follows: “hasn’t happened”, “once or twice”, “2–3 times a month”, “about once a week”, and “several times a week”. The participants who answered “once or twice” or more frequently to any of the items related to traditional victimisation were categorised as victims of traditional bullying. Similarly, the participants who answered “once or twice” or more frequently to any questions related to traditional bullying perpetration were categorised as bullies involved in traditional bullying. The participants who were simultaneously victims and bullies were categorised as bully-victims of traditional bullying. In this study, Cronbach’s alphas were 0.67 for victimisation and 0.71 for perpetration.

#### Cyberbullying

The questions related to cyberbullying were based on a previous study performed in Taiwan [27] and our qualitative study findings in 2016 [28]. The questionnaire included seven items related to cyberbullying victimisation and perpetration. The participants were asked how frequently they had experienced cyber victimisation in the previous 2 months: “How often has someone (1) made or posted rude comments to or about you online; (2) posted embarrassing pictures or videos of you online; (3) spread rumours about you online; (4) posted your personal information online; (5) insulted you publicly online; (6) made threatening comments to hurt you online; or (7) excluded or ignored you online on purpose?” Subsequently, they were asked how often they had perpetrated the above seven behaviours towards others. Each item was evaluated on a 5-point scale as follows: “hasn’t happened”, “once or twice”, “2–3 times a month”, “about once a week”, and “several times a week”. The participants who answered “once or twice” or more frequently to any questions related to cyber victimisation were categorised as victims of cyberbullying. Similarly, the participants who answered “once or twice” or more frequently to any questions related to cyberbullying perpetration were categorised as cyberbullies. The participants who were simultaneously victims and bullies were categorised as bully-victims involved in cyberbullying. Cronbach’s alphas were 0.70 for victimisation and 0.66 for perpetration.

#### Individual factors

##### Demographic factors

The participants were asked about their age and gender.

##### Academic level

To measure academic level, the participants were asked, “How do you rate your academic performance in class?” The responses included “the top few”, “above average”, “around average”, “below average”, and “I don’t know”. The responses were recoded into “above average” and “average or below average” for analysis.

##### Internet usage time

The participants were asked the following question: “On an average school day, how many hours do you play computer games or smartphone games or use a computer for something that is not school work? (Count time spent on devices such as an iPad or other tablet, a smartphone, texting apps, YouTube, Instagram, Facebook, LINE, WhatsApp, or other social media)” [30]. The responses included “0 hours per day”, “less than 1 hour per day”, “between 1 and 2 hours per day”, “between 2 and 3 hours per day”, “between 3 and 4 hours per day”, “between 4 and 5 hours per day”, and “more than 5 hours per day”. The responses were categorised as “less than 3 hours per day” or “3 hours or more per day” for the analysis.

##### Internet addiction

Young’s Internet Addiction Diagnostic Questionnaire [31] was adopted to measure internet addiction. This scale contains eight items answered with “Yes” or “No” responses. The responses were recorded as “no addiction” (0–2 items) or “addiction” (3–8 items). Kuder-Richardson formula 20 was 0.69 in this study.

##### Substance use (smoking and alcohol use)

The participants were asked the following questions: “During the past 30 days, on how many days did you smoke a cigarette?” [30] and “During the past 30 days, on how many days did you use alcohol?” The responses included “0 days”, “1 or 2 days”, “3 to 5 days”, “6 to 9 days”, “10 to 19 days”, “20 to 29 days”, and “All 30 days” [30]. The responses were recoded as “Yes” (other options) or “No” (0 days) for analysis.

##### Psychological and psychiatric factors—self-esteem, psychological distress, self-harm, and suicidal ideation

Self-esteem was assessed by the Rosenberg Self-Esteem Scale [32], which contains ten items that measure individuals’ self-esteem (Cronbach’s alpha was 0.87 in this study). The short version of the Kessler Psychological Distress Scale (six items) [33] was used to assess the psychological distress of the participants (Cronbach’s alpha was 0.82 in this study). To measure self-harm and suicidal ideation, the participants were asked the following questions: “Have you ever self-harmed in the past 30 days?” and “Have you ever seriously considered attempting suicide in the past 30 days?”

#### Family factors

##### Living situation

The participants were asked whether they lived with both parents. The responses included “Yes, living with both parents”, “No, living with a single parent”, or “No, living with others”.

##### Parental internet supervision

The participants were asked whether their parents or guardians supervised their internet use. The responses included “Yes” and “No”.

##### Dinner days with family

To examine how frequently they ate dinner with their parents, the participants were asked the following: “During an average week, how many days do you eat evening meals with your family (0 to 7 days)?” [34]. The responses were recorded as “0–4 times” or “5–7 times”.

#### School/social factors

##### Type of schools

The participants were recruited from academic high schools and vocational high schools.

##### School climate

The school climate was assessed using nine items from the California School Climate and Safety Survey (CSCSS) [12, 35, 39, 40]. Each item was evaluated on a 5-point Likert scale ranging from “strongly disagree” (1) to “strongly agree” (5). The total scores ranged from 9 to 45, with higher scores indicating a perception of a positive school climate. In this study, Cronbach’s alpha was 0.89.

##### Perceived social support

The Multidimensional Scale of Perceived Social Support (MSPSS) [36] was used in the current study. The MSPSS includes 12 items that measure perceived social support from family, friends, and a significant other. Each item was evaluated on a 7-point Likert scale ranging from “very strongly disagree” (1) to “very strongly agree” (7). The total scores were aggregated and then divided by 12. The scores ranged from 1 to 7, with higher scores indicating higher social support. Cronbach’s alpha was 0.91 in this study.

#### Data analyses

The data were statistically analysed using IBM SPSS Statistics software version 22 (PASW) for Windows (SPSS, Inc., Chicago, Illinois, USA). Univariate analysis was performed to examine the descriptive statistics (central tendency, dispersion, frequency distribution table, etc.) of all study variables.

#### Distribution of the participants by type of bullying and role in bullying

The participants were classified as bullies, victims, bully-victims, or uninvolved for cyberbullying and traditional bullying. Participants who had been involved in both types of bullying were grouped into the “combined bullying group”. The prevalence rates of cyberbullying, traditional bullying and their combination were assessed in this study.

#### Characteristics of participants by type of bullying and role in bullying

Since the characteristics of the bully-victims were much more similar to those of the bullies than those of the victims, bully-victims and bullies were collapsed into the category of “bullies/bully-victims”. The participants placed in the “involved group” represented students who had played any role in any type of bullying, as opposed to students who did not play any role in any type of bullying (uninvolved group). Bivariate analyses of the involved group and the uninvolved group were performed using the chi-square test.

#### Correlated profiles by the type of bullying and role in bullying

Multinomial logistic regressions were performed to examine the variables associated with bullying roles (with the uninvolved group as a reference). Considering the effects of clustering by schools and classrooms, univariate and multinomial logistic regressions were performed using the SPSS Complex Samples module to correct the estimates of standard errors and significance tests on the regression. In this study, missing values for each variable were less than 2%, and listwise deletion was performed to address missing data.

The analysis was performed in the following steps to build a model for multinomial logistic regression. First, univariate logistic regression was performed as a pre-selection strategy to test all variables. Variables associated with bullying (*P* level less than 0.2) were considered candidates for inclusion in multinomial logistic regressions [41]. In this step, age, self-esteem, academic level, living situation, parental internet supervision, and perceived social support were not significant at the 0.2 level.

Second, the first multinomial logistic regression was performed using all variables the identified by the first step. According to this model, the variables were examined for significance using the *P*-value (< 0.05) determine by Wald statistics [41]. Internet usage time (*P* = 0.265), smoking (*P* = 0.409), suicidal ideation (*P* = 0.086), and family dinner days (*P* = 0.165) were not significant at the 0.05 level. Considering that suicidal ideation is epidemiologically important [3, 42] to bullying, it was not eliminated from the final model.

Based on these selective strategies, gender, internet addiction, alcohol use, self-harm, suicidal ideation, types of school and school climate were included as independent variables in the final model. In addition, collinearity diagnostics were assessed among independent variables to take into account multicollinearity in the final model. The variance inflation factor (VIF) values were under two for all variables, indicating that multicollinearity was not a concern in the final model.

## Results

Of the 3270 eligible students, 2111 participated in the survey (response rate = 64.5%). Of the 2111 returned questionnaires, we excluded invalid questionnaires based on the following criteria: (1) response bias: the participants completed the questionnaires by filling in the same answer repeatedly or filling in answers according to a pattern; (2) incomplete questionnaires; and (3) missing values for the outcome variables: participants submitted questionnaires with incomplete information for the traditional bullying and cyberbullying items. Finally, 2028 questionnaires were analysed in this study, with an effective response rate of 62.0%.

### Prevalence and overlap of cyberbullying and traditional bullying

Overall, 32.6% (661/2028) of the participants reported they had been involved in bullying. The prevalence rates of cyberbullying and traditional bullying were 19.3% (392/2028) and 22.7% (460/2028), respectively. However, considering the overlap between the two types of bullying, the prevalence rates of cyberbullying, traditional bullying and combined bullying were 9.9% (201/2028), 13.3% (269/2028), and 9.4% (191/2028), respectively. In addition, 48.7% of the participants involved in cyberbullying had experienced traditional bullying, while 41.5% of those involved in traditional bullying had experienced cyberbullying.

### Distribution of the participants by type of bullying and role in bullying

The distributions of cyberbullying, traditional bullying, and combined bullying by bullying roles are shown in Table 1. Of those who were involved in bullying, 40.7% were involved traditional bullying, followed by cyberbullying (30.4%) and combined bullying (28.9%). Regarding the role in bullying, victims represented the largest population (47.9%), followed by bully-victims (34.6%) and bullies (17.5%). A similar pattern was observed for cyberbullying and traditional bullying; however, it was most prominent in cyberbullying, in which 67.7% (136/201) of the sample were victims. Similarly, of the participants involved in traditional bullying, 45.4% (122/269) were victims, followed by bully-victims (29.7%) and bullies (24.9%).

**Table 1:** Role distribution by type of bullying

Type of bullying	Bullying role			
	Victim	Bully	Bully-victim	Total
Cyberbullying	136 (20.6)	29 (4.4)	36 (5.4)	201 (30.4)
Traditional bullying	122 (18.5)	67 (10.1)	80 (12.1)	269 (40.7)
Combined bullying	58 (8.8)	20 (3.0)	113 (17.1)	191 (28.9)
Total	316 (47.9)	116 (17.5)	229 (34.6)	661 (100.0)

### Characteristics of participants by type of bullying and role in bullying

Table 2 describes the characteristics of participants by type-role categories of bullying.

**Table 2:** Distribution of individual, familial and school/social factors by type-role categories of bullying

Variables	Total	Traditional bullying		Cyberbullying		Combined bullying		Involved in any types of bullying	Uninvolved	*P-value*^*a*^
		Victims	Bullies/bully-victims	Victims	Bullies/bully-victims	Victims	Bullies/bully-victims			
	(*N* = 2028)	(*N* = 122)	(*N* = 147)	(*N* = 136)	(*N* = 65)	(*N* = 58)	(*N* = 133)	(*N* = 661)	(*N* = 1367)	
	n (%)	n (%)	n (%)	n (%)	n (%)	n (%)	n (%)	n (%)	n (%)	
Individual factors										
Age										
14–16	1290 (63.6)	82 (67.2)	105 (71.4)	89 (65.4)	38 (58.5)	34 (58.6)	81 (60.9)	429 (64.9)	861 (63.0)	0.446
17–20	731 (36.0)	40 (32.8)	42 (28.6)	46 (33.8)	27 (41.5)	24 (41.4)	52 (39.1)	231 (34.9)	500 (36.6)	
Missing	7 (0.4)			1 (0.7)				1 (0.2)	6 (0.4)	
Gender										
Male	979 (48.3)	61 (50.0)	101 (68.7)	53 (39.0)	37 (56.9)	17 (29.3)	77 (57.9)	346 (52.3)	633 (46.3)	0.012
Female	1043 (51.4)	61 (50.0)	45 (30.6)	83 (61.0)	28 (43.1)	41 (70.7)	56 (42.1)	314 (47.5)	729 (53.3)	
Missing	6 (0.3)		1 (0.7)					1 (0.2)	5 (0.4)	
Academic level										
Above average	875 (43.1)	49 (40.2)	74 (50.3)	53 (39.0)	26 (40.0)	25 (43.1)	50 (37.6)	277 (41.9)	598 (43.7)	0.425
Average or below	1152 (56.8)	73 (59.8)	73 (49.7)	83 (61.0)	39 (60.0)	33 (56.9)	83 (62.4)	384 (58.1)	768 (56.2)	
Missing	1 (0.1)								1 (0.1)	
Internet usage time per day										
< 3 h	1135 (56.0)	66 (54.1)	79 (53.7)	72 (52.9)	28 (43.1)	34 (58.6)	57 (42.9)	336 (50.8)	799 (58.4)	0.001
≥ 3 h	882 (43.5)	55 (45.1)	68 (46.3)	64 (47.1)	37 (56.9)	24 (41.4)	75 (56.4)	323 (48.9)	559 (40.9)	
Missing	11 (0.5)	1 (0.8)					1 (0.8)	2 (0.3)	9 (0.7)	
Internet addiction										
No addiction	881 (43.4)	51 (41.8)	55 (37.4)	55 (40.4)	27 (41.5)	13 (22.4)	33 (24.8)	234 (35.4)	647 (47.3)	< 0.001
Addiction	1147 (56.6)	71 (58.2)	92 (62.6)	81 (59.6)	38 (58.5)	45 (77.6)	100 (75.2)	427 (64.6)	720 (52.7)	
Smoking (past 30 days)										
No	1939 (95.6)	117 (95.9)	135 (91.8)	133 (97.8)	59 (90.8)	57 (98.3)	113 (85.0)	614 (92.9)	1325 (96.9)	< 0.001
Yes	85 (4.2)	4 (3.3)	12 (8.2)	3 (2.2)	6 (9.2)	0 (0.0)	19 (14.3)	44 (6.7)	41 (3.0)	
Missing	4 (0.2)	1 (0.8)				1 (1.7)	1 (0.8)	3 (0.5)	1 (0.1)	
Alcohol use (past 30 days)										
No	1510 (74.5)	98 (80.3)	92 (62.6)	93 (68.4)	42 (64.6)	41 (70.7)	69 (51.9)	435 (65.8)	1075 (78.6)	< 0.001
Yes	513 (25.3)	23 (18.9)	55 (37.4)	43 (31.6)	23 (35.4)	17 (29.3)	64 (48.1)	225 (34.0)	288 (21.1)	
Missing	5 (0.3)	1 (0.8)						1 (0.2)	4 (0.3)	
Self-esteem										
Low (< 15)	1504 (74.2)	100 (82.0)	111 (75.5)	103 (75.7)	50 (76.9)	46 (79.3)	101 (75.9)	511 (77.3)	993 (72.6)	0.009
Moderate to high (≥15)	493 (24.3)	21 (17.2)	30 (20.4)	32 (23.5)	14 (21.5)	10 (17.2)	29 (21.8)	136 (20.6)	357 (26.1)	
Missing	31 (1.5)	1 (0.8)	6 (4.1)	1 (0.7)	1 (1.5)	2 (3.5)	3 (2.3)	14 (2.1)	17 (1.2)	
Psychological distress										
No serious distress (< 13)	1400 (69.0)	73 (59.8)	84 (57.1)	78 (57.4)	44 (67.7)	32 (55.2)	67 (50.4)	378 (57.2)	1022 (74.8)	< 0.001
Yes (≥13)	628 (31.0)	49 (40.2)	63 (42.9)	58 (42.6)	21 (32.3)	26 (44.8)	66 (49.6)	283 (42.8)	345 (25.2)	
Self-harm										
No	1895 (93.4)	112 (91.8)	122 (83.0)	128 (94.1)	60 (92.3)	46 (79.3)	120 (90.2)	588 (89.0)	1307 (95.6)	< 0.001
Yes	132 (6.5)	10 (8.2)	25 (17.0)	8 (5.9)	5 (7.7)	12 (20.7)	13 (9.8)	73 (11.0)	59 (4.3)	
Missing	1 (0.1)								1 (0.1)	
Suicidal ideation										
No	1887 (93.0)	106 (86.9)	130 (88.4)	127 (93.4)	61 (93.8)	49 (84.5)	111 (83.5)	584 (88.4)	1303 (95.3)	< 0.001
Yes	141 (7.0)	16 (13.1)	17 (11.6)	9 (6.6)	4 (6.2)	9 (15.5)	22 (16.5)	77 (11.6)	64 (4.7)	
*Family factors*										
Living situation										
Both parents	1668 (82.2)	99 (81.1)	119 (81.0)	104 (76.5)	49 (75.4)	48 (82.8)	109 (82.0)	528 (79.9)	1140 (83.4)	0.092
Single parent	310 (15.3)	22 (18.0)	25 (17.0)	27 (19.9)	14 (21.5)	7 (12.1)	17 (12.8)	112 (16.9)	198 (14.5)	
With others	49 (2.4)	1 (0.8)	3 (2.0)	5 (3.7)	2 (3.1)	3 (5.2)	7 (5.3)	21 (3.2)	28 (2.1)	
Missing	1 (0.1)								1 (0.1)	
Parental internet supervision										
No	699 (34.5)	42 (34.4)	51 (34.7)	43 (31.6)	28 (43.1)	15 (25.9)	52 (39.1)	231 (34.9)	468 (34.2)	0.785
Yes	1319 (65.0)	79 (64.8)	96 (65.3)	93 (68.4)	37 (56.9)	43 (74.1)	80 (60.2)	428 (64.8)	891 (65.2)	
Missing	10 (0.5)	1 (0.8)					1 (0.8)	2 (0.3)	8 (0.6)	
Dinner days with family per week										
0–4 times	1078 (53.2)	65 (53.3)	84 (57.1)	78 (57.4)	42 (64.6)	35 (60.3)	91 (68.4)	395 (59.8)	683 (50.0)	< 0.001
5–7 times	938 (46.3)	56 (45.9)	62 (42.2)	58 (42.6)	23 (35.4)	23 (39.7)	40 (30.1)	262 (39.6)	676 (49.5)	
Missing	12 (0.6)	1 (0.8)	1 (0.7)				2 (1.5)	4 (0.6)	8 (0.6)	
*School/social factors*										
Types of school										
Academic	1573 (77.6)	94 (77.0)	87 (59.2)	116 (85.3)	47 (72.3)	51 (87.9)	91 (68.4)	486 (73.5)	1087 (79.5)	0.002
Vocational	455 (22.4)	28 (23.0)	60 (40.8)	20 (14.7)	18 (27.7)	7 (12.1)	42 (31.6)	175 (26.5)	280 (20.5)	
School climate										
Positive (> 32)	1491 (73.5)	85 (69.7)	98 (66.7)	99 (72.8)	41 (63.1)	32 (55.2)	72 (54.1)	427 (64.6)	1064 (77.8)	< 0.001
Negative (≤32)	537 (26.5)	37 (30.3)	49 (33.3)	37 (27.2)	24 (36.9)	26 (44.8)	61 (45.9)	234 (35.4)	303 (22.2)	
Perceived social support										
Low (1–2.9)	35 (1.7)	4 (3.3)	5 (3.4)	1 (0.7)	1 (1.5)	1 (1.7)	2 (1.5)	14 (2.1)	21 (1.5)	0.009
Moderate (3–5)	840 (41.4)	53 (43.4)	64 (43.5)	57 (41.9)	34 (52.3)	32 (55.2)	63 (47.4)	303 (45.8)	537 (39.3)	
High (5.1–7)	1153 (56.9)	65 (53.3)	78 (53.1)	78 (57.4)	30 (46.2)	25 (43.1)	68 (51.1)	344 (52.0)	809 (59.2)	

The total percentages may differ from 100 due to rounding

^a^*P*-values are from the chi-square test between the involved group and the uninvolved group

### Individual factors

The age of the participants ranged from 14 to 20 years, with a mean age of 16.2 (SD 0.71). Gender distribution varied according to the type-role category of bullying: the roles of victims of cyberbullying and combined bullying were dominated by women (61.0 and 70.7%, respectively), while the roles of bullies/bully-victims of traditional bullying were dominated by men (68.7%). Self-reported academic level was not related to any type-role category of bullying.

The time of internet use was generally greater among the students involved in all type-role categories of bullying compared to that of students who were uninvolved in bullying. The proportion of students using the internet 3 hours or more a day was significantly higher in the involved group (48.9%) than in the uninvolved group (40.9%). A similar pattern was observed for internet addiction, albeit with greater proportions (64.6% in the involved group vs. 52.7% in the uninvolved group).

Smoking was generally low among students (4.2% overall). However, there was a clear pattern showing that the proportion of students who smoked was higher (approximately 10%) in the bully/bully-victim groups for all types of bullying, while the proportion among victims ranged from 0 to 3.3%, close to or even lower than that of the uninvolved group (3.0%). Alcohol use was much more prevalent (25.3% overall) than smoking but showed a similar pattern of concentration, being more prevalent in the bully/bully-victim groups than in the victim groups.

Mental health problems were generally more prevalent in the involved group than in the uninvolved group. The proportions of students who experienced low self-esteem (below 15 points), serious psychological distress (13 points or more), self-harm, and suicidal ideation were all significantly greater in the involved group than the uninvolved group (77.3% vs. 72.6%, 42.8% vs. 25.2%, 11.0% vs. 4.3%, and 11.6% vs. 4.7%, respectively). Students with low self-esteem were most prevalent among the group of victims of traditional bullying, and serious psychological distress, self-harm, and suicidal ideation were most prevalent among the groups of combined bullying, except for self-harm in the bully/bully-victim group. Students experiencing self-harm and suicidal ideation were least prevalent among the cyberbullying groups of.

### Family factors

With regard to living situations and parental internet supervision, no significant difference was observed between the involved and uninvolved groups. However, the proportion of the students who had dinner with their family four times or less per week was generally greater among all type-role categories of bullying, with the difference between involved and uninvolved groups being statistically significant.

### School/social factors

Irrespective of the type of bullying, the proportion of vocational high school students in the bully/bully-victim group was at least twice as large as that than in the victim group. On the other hand, negative school climate (32 points or less) was generally more prevalent in all type-role categories of bullying (35.4% overall) than in the uninvolved group (22.2%), with a particular concentration (approximately 45%) among subgroups of combined bullying. The number of students with a high level of social support (5.1 points or more) was lower in all type-role categories of bullying (52.0% overall) than in the uninvolved group (59.2%).

### Correlate profiles by the type and role of bullying

Table 3 shows the results of the multinomial logistic regression analysis using the Complex Samples module of SPSS with the uninvolved group as a reference. From this analysis, being a victim of traditional bullying was found to be significantly associated with serious psychological distress (OR 1.74) and suicidal ideation (OR 2.34), suggesting that this type of bullying has a potent mental health impact on victims that may lead even to suicide. Being a bully/bully-victim of traditional bullying was also associated with psychological distress (OR 1.91) and self-harm (OR 4.01) as well as being male (OR 2.80), being addicted to the internet (OR 1.46), engaging in alcohol use (OR 1.88), being a vocational school student (OR 2.43), and having a negative school climate (OR 1.55) with statistical significance. Such findings suggested that students with such demographic and behavioural backgrounds and educational environments were more likely to be bullies/bully-victims of traditional bullying.

**Table 3:** Multivariate correlates by type-role category of bullying with uninvolved group as a reference

Variables	Traditional bullying				Cyberbullying				Combined bullying			
	Victims (N = 122)		Bullies/bully-victims (N = 147)		Victims (N = 136)		Bullies/bully-victims (N = 65)		Victims (N = 58)		Bullies/bully-victims (N = 133)	
	AOR (95% CI)	*P-value*	AOR (95% CI)	*P-value*	AOR (95% CI)	*P-value*	AOR (95% CI)	*P-value*	AOR (95% CI)	*P-value*	AOR (95% CI)	*P-value*
Gender												
Female	1 (ref)		1 (ref)		1 (ref)		1 (ref)		1 (ref)		1 (ref)	
Male	1.28 (0.84,1.95)	0.244	2.80 (2.00,3.91)	< 0.001	0.83 (0.54,1.30)	0.405	1.49 (0.90,2.47)	0.115	0.62 (0.32,1.18)	0.138	1.79 (1.17,2.73)	0.010
Internet addiction												
No addiction	1 (ref)		1 (ref)		1 (ref)		1 (ref)		1 (ref)		1 (ref)	
Addiction	1.17 (0.84,1.63)	0.325	1.46 (1.02,2.08)	0.041	1.15 (0.74,1.78)	0.529	1.28 (0.78,2.11)	0.319	2.60 (1.36,4.97)	0.006	2.61 (1.63,4.20)	< 0.001
Alcohol use												
No	1 (ref)		1 (ref)		1 (ref)		1 (ref)		1 (ref)		1 (ref)	
Yes	0.79 (0.52,1.21)	0.257	1.88 (1.33,2.64)	0.001	1.68 (1.13,2.50)	0.012	1.87 (1.09,3.22)	0.026	1.47 (0.86,2.50)	0.151	2.90 (1.98,4.26)	< 0.001
Psychological distress												
No serious distress	1 (ref)		1 (ref)		1 (ref)		1 (ref)		1 (ref)		1 (ref)	
Yes	1.74 (1.16,2.61)	0.009	1.91 (1.27,2.88)	0.003	1.98 (1.36,2.87)	0.001	1.27 (0.74,2.20)	0.368	1.25 (0.66,2.37)	0.476	2.08 (1.33,3.24)	0.003
Self-harm												
No	1 (ref)		1 (ref)		1 (ref)		1 (ref)		1 (ref)		1 (ref)	
Yes	1.20 (0.57,2.54)	0.615	4.01 (2.31,6.94)	< 0.001	0.98 (0.46,2.09)	0.951	1.78 (0.60,5.24)	0.282	3.55 (1.74,7.27)	0.001	1.24 (0.62,2.49)	0.525
Suicidal ideation												
No	1 (ref)		1 (ref)		1 (ref)		1 (ref)		1 (ref)		1 (ref)	
Yes	2.34 (1.28,4.27)	0.008	1.32 (0.70,2.49)	0.377	0.96 (0.46,2.00)	0.913	0.94 (0.33,2.66)	0.905	1.46 (0.56,3.83)	0.420	2.43 (1.41,4.18)	0.003
Type of school												
Academic	1 (ref)		1 (ref)		1 (ref)		1 (ref)		1 (ref)		1 (ref)	
Vocational	1.18 (0.71,1.96)	0.502	2.43 (1.52,3.90)	0.001	0.74 (0.43,1.29)	0.277	1.43 (0.81,2.52)	0.204	0.68 (0.28,1.67)	0.381	1.83 (1.10,3.06)	0.023
School climate												
Negative	1.41 (0.80,2.46)	0.220	1.55 (1.04,2.29)	0.032	1.19 (0.80,1.76)	0.384	1.95 (1.17,3.23)	0.012	2.69 (1.37,5.27)	0.006	2.58 (1.68,3.96)	< 0.001
Positive	1 (ref)		1 (ref)		1 (ref)		1 (ref)		1 (ref)		1 (ref)	

Notes: *AOR* adjusted odds ratio, *CI* confidence interval

In cyberbullying, being a victim was significantly associated with alcohol use (OR 1.68) and serious psychological distress (OR 1.98) but not with suicidal ideation, while being a bully/bully-victim was significantly associated with alcohol use (OR 1.87) and negative school climate (OR 1.95).

In combined bullying, being a victim was significantly associated with internet addiction (OR 2.60) and negative school climate (OR 2.69) and strongly associated with self-harm (OR 3.55), whereas being a bully/bully-victim showed, like traditional bullying, a broad correlate profile associated with psychological distress (OR 2.08) and suicidal ideation (OR 2.43) as well as with being male (OR 1.79), experiencing internet addiction (OR 2.61), using alcohol (OR 2.90), being a vocational school student (OR 1.83), and experiencing a negative school climate (OR 2.58). These findings suggest that the background/environmental factors of bullies/bully-victims are similar to those of traditional bullying, but that students in this role category likely have more serious mental health situations than those in the traditional bullying category.

## Discussion

This study provides updated information on the current situation of cyberbullying, traditional bullying, and their overlap (combined bullying) among Taiwanese high school students in Taipei. We compared the correlate profiles across the type-role categories of bullying from an ecological perspective for the first time in Asia, specifically Taiwan.

### Prevalence of cyberbullying and traditional bullying

Our study revealed that 9.9, 13.3, and 9.4% of the participants were involved in cyberbullying, traditional bullying, and combined bullying, respectively; that is, nearly one-third of all participants were involved in bullying. Additionally, when overlap (combined bullying) was considered, 19.3 and 22.7% of the participants experienced cyberbullying and traditional bullying, respectively. These prevalence rates and their relative predominance are similar to the results of a meta-analysis of 80 papers published between 2007 and 2013 [43], which showed that the prevalence rates were approximately 15 and 35% for cyberbullying and traditional bullying, respectively, for both perpetration and victimisation. In more recent studies conducted in East Asia, the pattern of relative predominance of traditional bullying over cyberbullying has also been reported in studies in China [44] and South Korea [45]. A study from China demonstrated that the prevalence rates of victimisation were 43.0 and 23.0% for traditional bullying and cyberbullying, respectively [44]. Furthermore, a Korean study showed that the prevalence rates of victimisation were 18.1, 12.8 and 3.5% for relational, verbal and physical bullying, respectively, and 5.6% for cyberbullying [45].

However, our results were not consistent with a previous study in Taipei in 2010 among 10th-grade high school students. That study reported that cyberbullying was more prevalent than traditional bullying (35.4% vs. 23.8%), though the prevalence of traditional bullying was similar to our data [27]. This discrepancy may be attributed to the difference in the years when the studies were conducted (2010 vs. 2018), the time frame of reference with regard to bullying experience (1 year vs. 2 months), the difference in response rates (80% vs. 62.0%), the partial difference in the question items about cyberbullying, or the decline in cyberbullying over time. The last possibility was suggested by a recent study in China that reported a decline in both cyberbullying and traditional bullying from 2016 to 2017 [44].

### Overlap between traditional bullying and cyberbullying

To date, only a few studies have reported the extent of overlap between cyberbullying and traditional bullying. Our study showed that 48.7% of the students involved in cyberbullying were also involved in traditional bullying, and 41.5% of those involved in traditional bullying were also involved in cyberbullying. Previous studies in Western societies have reported such an overlap to different extents. In the United States, among youth involved in cyberbullying, 36–56% experienced traditional bullying, depending on the role [18, 46]. Another study among high school students in the United States, albeit only among victims, showed that 59.7% of students involved in cyberbullying were also involved in traditional bullying, while 36.3% of students involved in traditional bullying were also involved in cyberbullying [9]. Other studies reported more pronounced overlap: over 80% of cyberbullied youth were also involved in traditional bullying in Norway and the United States [15, 19]. No information has been available about bullying overlap in East Asia to date, except for a study in Taiwan [27] that suggested the overlap only in the form of odds ratios. Possible reasons for the difference in the overlap rate may include socio-cultural differences in what people consider bullying. Whatever the reason, however, it is important to note that approximately half or more adolescents involved in bullying were involved in both types of bullying, which could lead to serious mental problems, as will be discussed later. The possibilities of such an overlap should be carefully examined when determining how to support adolescents involved in bullying.

### Role distribution in bullying

Our study showed that role distribution differs by the type of bullying. Though the most common role in all types of bullying was the victim, followed by the bully-victim role and then the bully role, this pattern was most prominent in cyberbullying, where victims filled two-thirds of the roles. Our results related to cyberbullying are consistent with the findings of previous studies [17, 27], probably reflecting the fact that compared with traditional bullying, a limited number of perpetrators can harm many people simultaneously in cyberbullying [8, 47, 48]. Regarding the role in bullying, it is also important to note that among the students involved in cyberbullying or traditional bullying, approximately 20–30% are bully-victims; that is, they had experienced both being a bully and a victim. This may mean that bullying is a dynamic phenomenon in which the role of bullying and victims can change over time depending on changes in the relative balance of power, as previously suggested [49, 50].

### Correlates of bullying

Though a direct comparison with previous studies is not possible because of the difference in the type and role classification of bullying, many of the major findings on the correlate profile of bullying are consistent with those of previous studies. For example, previous studies showed that traditional bullying victims were at high risk of suicidal ideation [51, 52], whereas cyberbullying victims were more likely to experience alcohol use [34] and psychological distress [53]. Regarding combined bullying victims, a region-wide study in the United States among high school students showed that they were at an enhanced risk of self-harm compared with victims of either traditional bullying or cyberbullying alone [9]. Their study also showed that victims of combined bullying were at an elevated risk of suicidal ideation and attempts, which was not identified in our study. Our study showed that bullies/bully-victims of traditional bullying were more likely to be involved in alcohol use and to have psychiatric problems. These findings may be related to a previous observation that adolescents who used alcohol [54] and had psychiatric problems [55] were more likely to be bullying perpetrators.

Notably, being a bully/bully-victim was associated with more specific background or environment in cases of traditional and combined bullying than in cyberbullying. Bullies/bully- victims in traditional and combined bullying were more likely to be male, have an internet addiction, drink, be students at a vocational school, and experience a negative school climate. An increased risk of vocational school students becoming bullies and bully-victims has also been reported in a previous study in Taiwan [56], suggesting that the peer environments of vocational schools may predispose students to bullying perpetration.

From these comparisons of correlate profiles between traditional and combined bullying, it can be suggested that bullying perpetration is more likely to occur among students who have behavioural problems and/or are in a specific school environment. Additionally, the similarities in these backgrounds between combined and traditional bullying suggest that combined bullying is more likely traditional bullying superimposed by cyberbullying rather than vice versa.

Regarding bullies and bully-victims of cyberbullying, several risk factors were identified in previous studies, including lower self-esteem [57, 58], internet addiction [59], depression [27, 58], suicidal ideation [60], and a low sense of belonging to school [25]. The numbers of bullies/bully-victims with internet addiction and a history of self-harm did not reach statistical significance, probably due to the limited sample size. In Taiwan, studies with larger sample sizes are clearly needed to obtain a clear correlate profile of bullies and bully-victims of cyberbullying.

Finally, it is important to note that both victims and bullies/bully-victims were strongly associated with mental or psychological problems. This suggests the importance of designing and developing prevention and support programmes addressing not only victims but also bullies and bully-victims to solve the problem of bullying among adolescents.

Our study has several limitations. First, its cross-sectional nature does not allow causal inferences or conclusions regarding the temporal sequence of the events to be drawn [61]. Second, since the response rate was only 62%, there could be selection bias between the students who participated and those who did not participate in the study. Third, as all data were self-reported by the students, social desirability bias and recall bias could not be avoided, though we made efforts to minimise them by making the questionnaire anonymous [62], allowing students to complete it at home to avoid peer pressure, and restricting the recall time frame to 2 months rather than 1 year. Fourth, and related to the response rate, the total sample size was not large enough to allow a sufficient sample size for subgroup analyses [17]. Fifth, although we developed a questionnaire based on our qualitative study and careful investigation of previous studies, our results were not directly comparable with those of other studies because of the difference in the items or time frame of questions on bullying. From these considerations, a standard questionnaire should be developed at least in the same country or within similar cultural settings. Additionally, a longitudinal study should be performed to make causal inferences and to clarify how bullying develops and overlaps over time with a large sample size that allows more precise subgroup analysis.

## Conclusions

This study showed that bullying is prevalent among high school students to the extent that as many as one-third of all students participating in the study were involved in traditional bullying, cyberbullying, and combined bullying. Not only victims but also bullies/bully-victims were associated with serious mental and psychological problems. It also suggested that bullying perpetration in traditional and combined bullying is associated with specific behavioural backgrounds and school environment.

These results may inform public policy and future prevention or support programmes for bullying among Taiwanese adolescents. First, greater priority should be given to measures to address bullying since one-third of all students are involved in it. Second, support should be provided not only to victims but also to bullies/bully-victims since both are associated with serious mental or psychological problems. Third, it should be assumed that bullying is a complex phenomenon since almost half of the students who are involved in one type of bullying are also involved in another. Fourth, students’ behavioural background and school environment should be taken into account for effective prevention of bullying.

## Supplementary information

**Additional file 1.** Questionnaire. The questionnaire which was developed for this study.

## Data Availability

The datasets generated and analysed during the current study are not publicly available because informed consent was not obtained for data sharing. Data are, however, available from the authors upon reasonable request.

## Abbreviations

AOR: Adjusted odds ratio
CI: Confidence interval
ICCs: Intraclass correlation coefficients
ICTs: Information and communication technologies
SD: Standard deviation
SNSs: Social networking services
VIF: Variance inflation factor

## References

[1] United Nations Educational, Scientific and Cultural Organization (UNESCO). School Violence and Bullying: Global Status Report, 13 January 2017. http://unesdoc.unesco.org/images/0024/002469/246970e.pdf. Accessed 18 Apr 2017.

[2] KALTIALA-HEINO RIITTAKERTTU, RIMPELÄ MATTI, RANTANEN PÄIVI, RIMPELÄ ARJA (2000). Bullying at school—an indicator of adolescents at risk for mental disorders. Journal of Adolescence.

[3] Holt M. K., Vivolo-Kantor A. M., Polanin J. R., Holland K. M., DeGue S., Matjasko J. L., Wolfe M., Reid G. (2015). Bullying and Suicidal Ideation and Behaviors: A Meta-Analysis. PEDIATRICS.

[4] Lenhart, A., Pew Research Center. (2015). Teens, Social Media & Technology Overview 2015. http://www.pewinternet.org/2015/04/09/teens-social-media-technology-2015/. Accessed 23 Apr 2018.

[5] Kessel Schneider S, O'Donnell L, Smith E (2015). Trends in cyberbullying and school bullying victimization in a regional census of high school students, 2006–-2012. J Sch Health..

[6] Livingstone S, Haddon L, Hasebrink U, Ólafsson K, O’Neill B, Smahel D, Staksrud E. EU Kids Online–Findings, methods, recommendations. LSE, London: EU Kids Online. http://lsedesignunit com/EUKidsOnline 2014. Accessed 10 Oct 2018.

[7] Smith PK, Steffgen G, Sittichai R, Smith PK, Steffgen G (2013). The nature of cyberbullying, and an international network. Cyberbullying through the new media: Findings from an international network.

[8] Patchin JW, Hinduja S (2006). Bullies move beyond the schoolyard a preliminary look at cyberbullying. Youth Violence Juv Justice..

[9] Schneider SK, O'donnell L, Stueve A, Coulter RW (2012). Cyberbullying, school bullying, and psychological distress: A regional census of high school students. Am J Public Health..

[10] Sourander A, Klomek AB, Ikonen M, Lindroos J, Luntamo T, Koskelainen M, Ristkari T, Helenius H (2010). Psychosocial risk factors associated with cyberbullying among adolescents: A population-based study. Arch Gen Psychiatry..

[11] Messias E, Kindrick K, Castro J (2014). School bullying, cyberbullying, or both: correlates of teen suicidality in the 2011 CDC Youth Risk Behavior Survey. Compr Psychiatry..

[12] Williams KR, Guerra NG (2007). Prevalence and predictors of internet bullying. J Adolesc Health..

[13] Cappadocia MC, Craig WM, Pepler D (2013). Cyberbullying: Prevalence, stability, and risk factors during adolescence. Can J Sch Psychol..

[14] Smith PK, Mahdavi J, Carvalho M, Fisher S, Russell S, Tippett N (2008). Cyberbullying: Its nature and impact in secondary school pupils. J Child Psychol Psychiatry..

[15] Olweus D (2012). Cyberbullying: An overrated phenomenon?. Eur J Dev Psychol..

[16] Dehue F, Bolman C, Vollink T, Pouwelse M (2012). Cyberbullying and traditional bullying in relation to adolescents’ perception of parenting. J Cyber Ther Rehabil.

[17] Kubiszewski V, Fontaine R, Potard C, Auzoult L (2015). Does cyberbullying overlap with school bullying when taking modality of involvement into account?. Comput Human Behav..

[18] Ybarra ML, Diener-West M, Leaf PJ (2007). Examining the overlap in Internet harassment and school bullying: Implications for school intervention. J Adolesc Health..

[19] Raskauskas J, Stoltz AD (2007). Involvement in traditional and electronic bullying among adolescents. Dev Psychol..

[20] Mishna F, Khoury-Kassabri M, Gadalla T, Daciuk J (2012). Risk factors for involvement in cyber bullying: Victims, bullies and bully–victims. Child Youth Serv Rev..

[21] Fanti KA, Demetriou AG, Hawa VV (2012). A longitudinal study of cyberbullying: Examining risk and protective factors. Eur J Dev Psychol..

[22] Låftman SB, Modin B, Östberg V (2013). Cyberbullying and subjective health: A large-scale study of students in Stockholm. Sweden. Child Youth Serv Rev..

[23] Papatraianou LH, Levine D, West D (2014). Resilience in the face of cyberbullying: An ecological perspective on young people’s experiences of online adversity. Pastor Care Educ..

[24] Zhou Z, Tang H, Tian Y, Wei H, Zhang F, Morrison CM (2013). Cyberbullying and its risk factors among Chinese high school students. Sch Psychol Int..

[25] Wong DS, Chan HCO, Cheng CH (2014). Cyberbullying perpetration and victimization among adolescents in Hong Kong. Child Youth Serv Rev..

[26] Lee C, Shin N (2017). Prevalence of cyberbullying and predictors of cyberbullying perpetration among Korean adolescents. Comput Human Behav..

[27] Chang FC, Lee CM, Chiu CH, Hsi WY, Huang TF, Pan YC (2013). Relationships among cyberbullying, school bullying, and mental health in Taiwanese adolescents. J Sch Health..

[28] Wang C-W, Musumari PM, Techasrivichien T, Suguimoto SP, Chan C-C, Ono-Kihara M, Kihara M (2019). Nakayama T: “I felt angry, but I couldn’t do anything about it”: a qualitative study of cyberbullying among Taiwanese high school students. BMC public health..

[29] Olweus D. The revised Olweus bully/victim questionnaire: University of Bergen, Research Center for Health Promotion; 1996.

[30] Center for Disease Control and Prevention. 2017 State and Local Youth Risk Behavior Survey Questionnaire. https://www.cdc.gov/healthyyouth/data/yrbs/pdf/2017/2017_yrbs_standard_hs_questionnaire.pdf. Accessed May 31 2017.

[31] Young KS (2004). Internet addiction. A new clinical phenomenon and its consequences. Am Behav Sci..

[32] Rosenberg M. Society and the adolescent self-image. 1965. 10.1515/9781400876136.

[33] Kessler RC, Barker PR, Colpe LJ, Epstein JF, Gfroerer JC, Hiripi E, Howes MJ, Normand S-LT, Manderscheid RW, Walters EE (2003). Screening for serious mental illness in the general population. Arch Gen Psychiatry..

[34] Elgar FJ, Napoletano A, Saul G, Dirks MA, Craig W, Poteat VP, Holt M, Koenig BW (2014). Cyberbullying victimization and mental health in adolescents and the moderating role of family dinners. JAMA Pediatr..

[35] Furlong MJ, Greif JL, Bates MP, Whipple AD, Jimenez TC, Morrison R (2005). Development of the California school climate and safety survey-short form. Psychol Sch..

[36] Zimet GD, Dahlem NW, Zimet SG, Farley GK (1988). The multidimensional scale of perceived social support. J Pers Assess..

[37] Landis J. Richard, Koch Gary G. (1977). The Measurement of Observer Agreement for Categorical Data. Biometrics.

[38] Cicchetti DV (1994). Guidelines, criteria, and rules of thumb for evaluating normed and standardized assessment instruments in psychology. Psychol Assess..

[39] Wei H-S, Williams JH, Chen J-K, Chang H-Y (2010). The effects of individual characteristics, teacher practice, and school organizational factors on students' bullying: A multilevel analysis of public middle schools in Taiwan. Child Youth Serv Rev..

[40] Astor RA, Benbenishty R, Vinokur AD, Zeira A (2006). Arab and Jewish elementary school students' perceptions of fear and school violence: Understanding the influence of school context. Br J Educ Psychol..

[41] Hosmer David W., Lemeshow Stanley, Sturdivant Rodney X. (2013). Applied Logistic Regression.

[42] Klomek AB, Marrocco F, Kleinman M, Schonfeld IS, Gould MS (2007). Bullying, depression, and suicidality in adolescents. J Am Acad Child Adolesc Psychiatry..

[43] Modecki KL, Minchin J, Harbaugh AG, Guerra NG, Runions KC (2014). Bullying prevalence across contexts: A meta-analysis measuring cyber and traditional bullying. J Adolesc Health..

[44] Chu Xiao-Wei, Fan Cui-Ying, Liu Qing-Qi, Zhou Zong-Kui (2018). Stability and Change of Bullying Roles in the Traditional and Virtual Contexts: A Three-Wave Longitudinal Study in Chinese Early Adolescents. Journal of Youth and Adolescence.

[45] Yun I, Kim S-G (2016). Bullying among South Korean adolescents: prevalence and association with psychological adjustment. Violence Vict..

[46] Ybarra ML, Mitchell KJ (2004). Online aggressor/targets, aggressors, and targets: A comparison of associated youth characteristics. J Child Psychol Psychiatry..

[47] Ovejero Anastasio, Yubero Santiago, Larrañaga Elisa, de la V. Moral María (2015). Cyberbullying: Definitions and Facts from a Psychosocial Perspective. Cyberbullying Across the Globe.

[48] Kowalski RM, Giumetti GW, Schroeder AN, Lattanner MR (2014). Bullying in the digital age: A critical review and meta-analysis of cyberbullying research among youth. Psychol Bull..

[49] Barker ED, Arseneault L, Brendgen M, Fontaine N, Maughan B (2008). Joint development of bullying and victimization in adolescence: Relations to delinquency and self-harm. J Am Acad Child Adolesc Psychiatry..

[50] Haltigan JD, Vaillancourt T (2014). Joint trajectories of bullying and peer victimization across elementary and middle school and associations with symptoms of psychopathology. Dev Psychol..

[51] Geoffroy M-C, Boivin M, Arseneault L, Turecki G, Vitaro F, Brendgen M, Renaud J, Séguin JR, Tremblay RE, Côté SM (2016). Associations between peer victimization and suicidal ideation and suicide attempt during adolescence: results from a prospective population-based birth cohort. J Am Acad Child Adolesc Psychiatry..

[52] Sampasa-Kanyinga H, Roumeliotis P, Xu H (2014). Associations between cyberbullying and school bullying victimization and suicidal ideation, plans and attempts among Canadian schoolchildren. PLoS One..

[53] Kim Soyeon, Kimber Melissa, Boyle Michael H., Georgiades Katholiki (2018). Sex Differences in the Association Between Cyberbullying Victimization and Mental Health, Substance Use, and Suicidal Ideation in Adolescents. The Canadian Journal of Psychiatry.

[54] Radliff KM, Wheaton JE, Robinson K, Morris J (2012). Illuminating the relationship between bullying and substance use among middle and high school youth. Addict Behav..

[55] Kumpulainen K, Räsänen E, Puura K (2001). Psychiatric disorders and the use of mental health services among children involved in bullying. Aggress Behav.

[56] Chen J-K, Avi AR (2009). The perpetration of school violence in Taiwan: An analysis of gender, grade level and school type. Sch Psychol Int.

[57] Patchin JW, Hinduja S (2010). Cyberbullying and self-esteem*. J Sch Health..

[58] Yang S-J, Stewart R, Kim J-M, Kim S-W, Shin I-S, Dewey ME, Maskey S, Yoon J-S (2013). Differences in predictors of traditional and cyber-bullying: a 2-year longitudinal study in Korean school children. Eur Child Adolesc Psychiatry.

[59] Chang F-C, Chiu C-H, Miao N-F, Chen P-H, Lee C-M, Chiang J-T, Pan Y-C (2015). The relationship between parental mediation and Internet addiction among adolescents, and the association with cyberbullying and depression. Compr Psychiatry.

[60] Hinduja S, Patchin JW (2010). Bullying, cyberbullying, and suicide. Arch Suicide Res..

[61] Mann C (2003). Observational research methods. Research design II: cohort, cross sectional, and case-control studies. Emerg Med J.

[62] Joinson A (1999). Social desirability, anonymity, and Internet-based questionnaires. Behav Res Methods Instrum Comput..

