# Supplementary material for: Overlap of traditional bullying and cyberbullying and correlates of bullying among Taiwanese adolescents: a cross-sectional study
Source: BMC Public Health. 2019 Dec 30;19:1756. doi: 10.1186/s12889-019-8116-z (PMC6937625; doi:10.1186/s12889-019-8116-z)
Supplement: Supplementary file 1 — Additional file 1. Questionnaire. The questionnaire which was developed for this study. [file 12889_2019_8116_MOESM1_ESM.docx]

2018

**Survey of school life, internet usage and mental health among senior high school students in Taiwan**

Due to the increased use of the internet over past 20 years, there has been growing concern about the health implications of internet use among adolescents. This research is being conducted to understand school life, internet usage and mental health among senior high school students in Taiwan.

The results of the questionnaire will read like this: “**How many people have this particular opinion**?” “**How many people have this particular experience**?”, showing only the number of people or percentage (%). This questionnaire is anonymous; it will be impossible for anyone to know your answers.

Your cooperation is really valuable to us. All answers from every senior high student are very important to us. Your answers will be kept private. Please don’t worry, and please answer this questionnaire honestly.

Thank you very much.

Sincerely appreciate your cooperation.

**【Research investigators】**

| Chia-Wen Wang  School of Public Health, Faculty of Medicine  Kyoto University, Japan | Chang-Chuan Chan, Professor  Global Health Center, College of Public Health, National Taiwan University |
| --- | --- |

**【Co-organizer】**

Department of Education, Taipei City Government

- Please seal your questionnaire with the sticker provided. You don’t need to write your name on the questionnaire.
- If you have any questions about the questionnaire, please contact us. Our contact information is provided on the last page.

|  | **About your background** | | | |
| --- | --- | --- | --- | --- |
|  |  | | | |
| Q 1) | How old are you? | | | |
|  | \|  \| \| --- \|   years old | | | |
| Q 2) | What grade are you in? | | | |
|  | 1.☐ | 10^th^ grade | | |
|  | 2.☐ | 11^th^ grade | | |
| Q 3) | What is your gender? | | | |
|  | 1.☐ | | | Male |
|  | 2.☐ | | | Female |
| Q 4) | How do you rate your academic performance in class? | | | |
|  | 1.☐ | | The top few | |
|  | 2.☐ | | Above average | |
|  | 3.☐ | | Around average | |
|  | 4.☐ | | Below average | |
|  | 5.☐ | | I don’t know | |
| Q 5) | Do you live with your both parents? | | | |
|  | 1.☐ | | Yes, living with both parents. | |
|  | 2.☐ | | No, living with a single parent. | |
|  | 3.☐ | | No, living with others | |

| Q 6) | On an average school day, how many hours do you play computer games or smartphone games or use a computer for something that is not school work?  **(Count time spent on devices such as an iPad or other tablet, a smartphone, texting apps, YouTube, Instagram, Facebook, LINE, WhatsApp, or other social media)** | | | |
| --- | --- | --- | --- | --- |
|  | 1.☐ | 0 hours per day | | |
|  | 2.☐ | Less than 1 hour per day | | |
|  | 3.☐ | Between 1 and 2 hours per day | | |
|  | 4.☐ | Between 2 and 3 hours per day | | |
|  | 5.☐ | Between 3 and 4 hours per day | | |
|  | 6.☐ | Between 4 and 5 hours per day | | |
|  | 7.☐ | More than 5 hours per day | | |
| Q 7) | Do your parents or guardians supervise your internet use? | | | |
|  | 1.☐ | Yes | | |
|  | 2.☐ | No | | |
| Q 8) | During an average week, how many days do you eat evening meals with your family? | | | |
|  | 1.☐ | 0 days |  |  |
|  | 2.☐ | 1 day |  |  |
|  | 3.☐ | 2 days |  |  |
|  | 4.☐ | 3 days |  |  |
|  | 5.☐ | 4 days |  |  |
|  | 6.☐ | 5 days |  |  |
|  | 7.☐ | 6 days |  |  |
|  | 8.☐ | 7 days |  |  |

| **Below is a list of statements regarding your general feelings about yourself.** | | | | | |  |
| --- | --- | --- | --- | --- | --- | --- |
| Q 9) | How often have you felt or behaved in the following ways? | | | | |  |
|  | |  |  |  |  |  |
|  | | **Strongly**  **disagree** | **Disagree** | **Agree** | **Strongly**  **agree** |  |
| 1. On the whole, I am satisfied with myself. | | | 1.☐ | 2.☐ | 3.☐ | 4.☐ |
| 1. At times, I think I am no good at all. | | | 1.☐ | 2.☐ | 3.☐ | 4.☐ |
| 1. I feel that I have a number of good qualities. | | | 1.☐ | 2.☐ | 3.☐ | 4.☐ |
| 1. I am able to do things as well as most other people. | | | 1.☐ | 2.☐ | 3.☐ | 4.☐ |
| 1. I feel that I do not have much to be proud of. | | | 1.☐ | 2.☐ | 3.☐ | 4.☐ |
| 1. I certainly feel useless at times. | | | 1.☐ | 2.☐ | 3.☐ | 4.☐ |
| 1. I feel that I am a person of worth, at least on an equal plane with others. | | | 1.☐ | 2.☐ | 3.☐ | 4.☐ |
| 1. I wish I could have more respect for myself. | | | 1.☐ | 2.☐ | 3.☐ | 4.☐ |
| 1. All in all, I am inclined to think that I am a failure. | | | 1.☐ | 2.☐ | 3.☐ | 4.☐ |
| 1. I take a positive attitude towards myself. | | | 1.☐ | 2.☐ | 3.☐ | 4.☐ |

**About your life experiences**

|  |  | | | |
| --- | --- | --- | --- | --- |
| Q 10) | Have you ever tried smoking cigarettes? | | | |
|  | 1.☐ | Yes | | |
|  | 2.☐ | No | | |
| Q 11) | During the past 30 days, on how many days did you smoke a cigarette? | | | |
|  | 1.☐ | 0 days |  |  |
|  | 2.☐ | 1 or 2 days |  |  |
|  | 3.☐ | 3 to 5 days |  |  |
|  | 4.☐ | 6 to 9 days |  |  |
|  | 5.☐ | 10 to 19 days |  |  |
|  | 6.☐ | 20 to 29 days |  |  |
|  | 7.☐ | All 30 days |  |  |
| Q 12) | Have you ever tried drinking alcohol? | | | |
|  | 1.☐ | Yes | | |
|  | 2.☐ | No | | |
| Q 13) | During the past 30 days, on how many days did you use alcohol? | | | |
|  | 1.☐ | 0 days |  |  |
|  | 2.☐ | 1 or 2 days |  |  |
|  | 3.☐ | 3 to 5 days |  |  |
|  | 4.☐ | 6 to 9 days |  |  |
|  | 5.☐ | 10 to 19 days |  |  |
|  | 6.☐ | 20 to 29 days |  |  |
|  | 7.☐ | All 30 days |  |  |

| Q 14) | During the past 30 days, about how often did you feel … | | | | | | | | |
| --- | --- | --- | --- | --- | --- | --- | --- | --- | --- |
|  | | | | | All  the  time | Most of the time | Some of the time | A little of the time | None of the time |
| 1. Nervous | | | | | 1.☐ | 2.☐ | 3.☐ | 4.☐ | 5.☐ |
| 1. Hopeless | | | | | 1.☐ | 2.☐ | 3.☐ | 4.☐ | 5.☐ |
| 1. Restless or fidgety | | | | | 1.☐ | 2.☐ | 3.☐ | 4.☐ | 5.☐ |
| 1. So depressed that nothing could cheer you up | | | | | 1.☐ | 2.☐ | 3.☐ | 4.☐ | 5.☐ |
| 1. That everything was an effort | | | | | 1.☐ | 2.☐ | 3.☐ | 4.☐ | 5.☐ |
| 1. Worthless | | | | | 1.☐ | 2.☐ | 3.☐ | 4.☐ | 5.☐ |
|  | |  | | | | | | | |
|  | |  | | | | | | | |
| Q 15) | | Have you ever self-harmed in the past 30 days (such as scratching, cutting, burning, or hitting yourself or banging your hand on purpose)? | | | | | | | |
|  | | 1.☐ | | Yes | | | | | |
|  | | 2.☐ | | No | | | | | |
|  | |  | |  | | | | | |
| Q 16) | | Have you ever seriously considered attempting suicide in the past 30 days? | | | | | | | |
|  | | 1.☐ | Yes | | | | | | |
|  | | 2.☐ | No | | | | | | |

**About your family and friends**

|  | |
| --- | --- |
| Q 17) | How do you feel about yourself and your family? |

|  | Very strongly disagree | Strongly disagree | Mildly disagree | Neutral | Mildly agree | Strongly agree | Very strongly agree |
| --- | --- | --- | --- | --- | --- | --- | --- |
| 1. My family really tries to help me. | 1.☐ | 2.☐ | 3.☐ | 4.☐ | 5.☐ | 6.☐ | 7.☐ |
| 1. I get the emotional help and support I need from my family. | 1.☐ | 2.☐ | 3.☐ | 4.☐ | 5.☐ | 6.☐ | 7.☐ |
| 1. I can talk about my problems with my family. | 1.☐ | 2.☐ | 3.☐ | 4.☐ | 5.☐ | 6.☐ | 7.☐ |
| 1. My family is willing to help me make decisions. | 1.☐ | 2.☐ | 3.☐ | 4.☐ | 5.☐ | 6.☐ | 7.☐ |

|  | Very strongly disagree | Strongly disagree | Mildly disagree | Neutral | Mildly agree | Strongly agree | Very strongly agree |
| --- | --- | --- | --- | --- | --- | --- | --- |
| 1. My friends really try to help me. | 1.☐ | 2.☐ | 3.☐ | 4.☐ | 5.☐ | 6.☐ | 7.☐ |
| 1. I can count on my friends when things go wrong. | 1.☐ | 2.☐ | 3.☐ | 4.☐ | 5.☐ | 6.☐ | 7.☐ |
| 1. I have friends with whom I can share my joys and sorrows. | 1.☐ | 2.☐ | 3.☐ | 4.☐ | 5.☐ | 6.☐ | 7.☐ |
| 1. I can talk about my problems with my friends. | 1.☐ | 2.☐ | 3.☐ | 4.☐ | 5.☐ | 6.☐ | 7.☐ |

| Q 18) | How do you feel about yourself and your friends? |
| --- | --- |
|  |  |

| Q 19) | How do you feel about yourself and other significant people in your life?    *a significant other might be you teacher, boyfriend/girlfriend, neighbour etc.—someone (except friends and family members) who is very important to you. |
| --- | --- |

|  | Very strongly disagree | Strongly disagree | Mildly disagree | Neutral | Mildly agree | Strongly agree | Very strongly agree |
| --- | --- | --- | --- | --- | --- | --- | --- |
| 1. There is a special person who is around when I am in need. | 1.☐ | 2.☐ | 3.☐ | 4.☐ | 5.☐ | 6.☐ | 7.☐ |
| 1. There is a special person with whom I can share my joys and sorrows. | 1.☐ | 2.☐ | 3.☐ | 4.☐ | 5.☐ | 6.☐ | 7.☐ |
| 1. I have a special person who is a real source of comfort to me. | 1.☐ | 2.☐ | 3.☐ | 4.☐ | 5.☐ | 6.☐ | 7.☐ |
| 1. There is a special person in my life who cares about my feelings. | 1.☐ | 2.☐ | 3.☐ | 4.☐ | 5.☐ | 6.☐ | 7.☐ |

| Q 20) | About your feelings when you use the internet; please answer the following questions. |
| --- | --- |

|  | Yes | No |
| --- | --- | --- |
| 1. Do you feel preoccupied with the internet?   (You are always thinking about previous online activity or anticipating your next online session.) | 1.☐ | 2.☐ |
| 1. Do you feel the need to use the internet for increasing amounts of time in order to achieve satisfaction? | 1.☐ | 2.☐ |
| 1. Have you repeatedly made unsuccessful efforts to control, cut back on, or stop your internet use? | 1.☐ | 2.☐ |
| 1. Do you feel restless, moody, depressed, or irritable when attempting to cut down on or stop your internet use? | 1.☐ | 2.☐ |
| 1. Do you sometimes stay online longer than you originally intended? | 1.☐ | 2.☐ |
| 1. Have you jeopardized or risked the loss of a significant relationship or been late to, left early from, or had an absence from school because of the internet? | 1.☐ | 2.☐ |
| 1. Have you lied to family members, your therapist, or others to conceal the extent of your involvement with the internet? | 1.☐ | 2.☐ |
| 1. Do you use the internet as a way to escape from problems or to relieve a dysphoric mood?   (e.g., Feelings of helplessness, guilt, anxiety, and depression) | 1.☐ | 2.☐ |

**About your school**

| Q 21) | Think about the way your school is most of the time. |
| --- | --- |

|  | Strongly  disagree | Disagree | Both agree and disagree | Agree | Strongly agree |
| --- | --- | --- | --- | --- | --- |
| 1. My teachers respect me. | 1.☐ | 2.☐ | 3.☐ | 4.☐ | 5.☐ |
| 1. My teachers are fair. | 1.☐ | 2.☐ | 3.☐ | 4.☐ | 5.☐ |
| 1. The teachers here are nice people. | 1.☐ | 2.☐ | 3.☐ | 4.☐ | 5.☐ |
| 1. When students break rules at my school, they are treated fairly. | 1.☐ | 2.☐ | 3.☐ | 4.☐ | 5.☐ |
| 1. The principal asks students about their ideas at my school. | 1.☐ | 2.☐ | 3.☐ | 4.☐ | 5.☐ |
| 1. My school is good place to be. | 1.☐ | 2.☐ | 3.☐ | 4.☐ | 5.☐ |
| 1. I feel like I belong at my school. | 1.☐ | 2.☐ | 3.☐ | 4.☐ | 5.☐ |
| 1. My school is important to me. | 1.☐ | 2.☐ | 3.☐ | 4.☐ | 5.☐ |
| 1. Teachers and staff at my school are doing the right things to prevent bullying. | 1.☐ | 2.☐ | 3.☐ | 4.☐ | 5.☐ |

**About your experiences**

|  |  | | | | | | |
| --- | --- | --- | --- | --- | --- | --- | --- |
| Q 22) | During the past two months, how often have other student(s)…  ***In real life, **not on the internet.** | | | | | | |
|  |  |  | **Has not happened** | **Once or twice** | **2-3 times a month** | **Once a week** | **Several times a week** |
|  | Called you mean names, made fun of you, or teased you in hurtful way? | | 1.☐ | 2.☐ | 3.☐ | 4.☐ | 5.☐ |
|  | Excluded you from their group of friends, or completely ignored you? | | 1.☐ | 2.☐ | 3.☐ | 4.☐ | 5.☐ |
|  | Hit, kicked, pushed or shoved you around? | | 1.☐ | 2.☐ | 3.☐ | 4.☐ | 5.☐ |
|  | Told lies or spread false rumours about you and tried to make others dislike you? | | 1.☐ | 2.☐ | 3.☐ | 4.☐ | 5.☐ |

| Q 23) | During the past two months, how often have you…  ***In real life, **not on the internet.** | | | | | |
| --- | --- | --- | --- | --- | --- | --- |
|  |  | **Has not happened** | **Once or twice** | **2-3 times a month** | **Once a week** | **Several times a week** |
|  | Called another student or multiple other students mean names and made fun of or teased them in hurtful way? | 1.☐ | 2.☐ | 3.☐ | 4.☐ | 5.☐ |
|  | Excluded them from your group of friends or completely ignored them? | 1.☐ | 2.☐ | 3.☐ | 4.☐ | 5.☐ |
|  | Hit, kicked, pushed, or shoved them around? | 1.☐ | 2.☐ | 3.☐ | 4.☐ | 5.☐ |
|  | Spread false rumours about them and tried to make others dislike them? | 1.☐ | 2.☐ | 3.☐ | 4.☐ | 5.☐ |

| Q 24) | During the past two months, how often has someone …  **[count your cyber experiences through instant messaging (LINE, Facebook Messenger, WhatsApp, WeChat, etc.), Instagram, Weibo, AZAR, Metero, Facebook (including your own school-related liked pages) or other social media or through online games** | | | | | |
| --- | --- | --- | --- | --- | --- | --- |
|  |  | **Has not happened** | **Once or twice** | **2-3 times a month** | **Once a week** | **Several times a week** |
|  | Made or posted rude comments to or about you online? | 1.☐ | 2.☐ | 3.☐ | 4.☐ | 5.☐ |
|  | Posted embarrassing pictures or videos of you online? | 1.☐ | 2.☐ | 3.☐ | 4.☐ | 5.☐ |
|  | Spread rumours about you online? | 1.☐ | 2.☐ | 3.☐ | 4.☐ | 5.☐ |
|  | Posted your personal information online? | 1.☐ | 2.☐ | 3.☐ | 4.☐ | 5.☐ |
|  | Insulted you publicly online? | 1.☐ | 2.☐ | 3.☐ | 4.☐ | 5.☐ |
|  | Made threatening comments to hurt you online? | 1.☐ | 2.☐ | 3.☐ | 4.☐ | 5.☐ |
|  | Excluded or ignored you online on purpose? | 1.☐ | 2.☐ | 3.☐ | 4.☐ | 5.☐ |

| Q 25) | During the past two months, how often have you…  **[count your cyber experiences through instant messaging (LINE, Facebook Messenger, WhatsApp, WeChat, etc.), Instagram, Weibo, AZAR, Metero, Facebook (including your own school-related liked pages) or other social media or through online games]** | | | | | |
| --- | --- | --- | --- | --- | --- | --- |
|  |  | **Has not happened** | **Once or twice** | **2-3 times a month** | **Once a week** | **Several times a week** |
|  | Made or posted rude comments to or about someone online? | 1.☐ | 2.☐ | 3.☐ | 4.☐ | 5.☐ |
|  | Posted embarrassing pictures or videos of someone online? | 1.☐ | 2.☐ | 3.☐ | 4.☐ | 5.☐ |
|  | Spread rumours about someone online? | 1.☐ | 2.☐ | 3.☐ | 4.☐ | 5.☐ |
|  | Posted someone’s personal information online? | 1.☐ | 2.☐ | 3.☐ | 4.☐ | 5.☐ |
|  | Insulted someone publicly online? | 1.☐ | 2.☐ | 3.☐ | 4.☐ | 5.☐ |
|  | Made threatening comments to hurt someone online? | 1.☐ | 2.☐ | 3.☐ | 4.☐ | 5.☐ |
|  | Excluded or ignored someone online on purpose? | 1.☐ | 2.☐ | 3.☐ | 4.☐ | 5.☐ |

**About your future**

Q 26) Lastly, if you wish people around you would do something, please write it down. Please also write about your future goals.

1. I wish my friends…
2. I wish my parents…
3. I wish the teachers….
4. My future goals…

**Please check questions 1 to 25 for any missing answers.**

**Thank you for your cooperation.**

**【Research investigators】**

Chia-Wen Wang

Principal Investigator

School of Public Health, Faculty of Medicine

Kyoto University, Japan

Email: am10312002 @gmail.com

Chang-Chuan Chan, Professor

Global Health Center, College of Public Health,

National Taiwan University, Taiwan

Tel: 02-3366-8094
